# 

**Published:** 1887-06

**Authors:** 


CONTENTS.

Vol. 34 No. 6.

| | PAGE. |
|---|---|
| Phrenology | 121 |
| Samuel Hahnemann | 124 |
| Transplanting an Eye | 129 |
| How a Star is weighed | 134 |
| Pre-Digested Foods | 137 |
| A wonderful Medium | 138 |
| A Cure for Consumption | 139 |
| The Voice of a Friend | 140 |
| Book Notices | 140 |
| Miscellaneous | 141 |
| Household | 143 |

| INDEX TO ADVERTISEMENTS OF HALF A PAGE AND OVER. | PAGE. |
|---|---|
| Winchester's Hypophosphate | I |
| Hollow Suppositories | II |
| Celerina | III |
| Lactopeptine | IV |
| Castoria | IV |
| Horsford's Acid Phosphate | V |
| Goodyear Rubber Co. | V |
| Crystalline Phosphate | VI |
| Bovinine | VII |
| Ten Valuable Books Free | VIII |
| Lactated Food | 2d page cover |
| " " | 3d page cover |

